# Environmental Cues Contribute to Dynamic Plasma Membrane Organization of Nanodomains Containing Flotillin-1 and Hypersensitive Induced Reaction-1 Proteins in *Arabidopsis thaliana*

**DOI:** 10.3389/fpls.2022.897594

**Published:** 2022-05-10

**Authors:** Changwen Xu, Sammar Abbas, Hongping Qian, Meng Yu, Xi Zhang, Xiaojuan Li, Yaning Cui, Jinxing Lin

**Affiliations:** ^1^National Engineering Research Center of Tree Breeding and Ecological Restoration, Beijing Forestry University, Beijing, China; ^2^College of Biological Sciences and Biotechnology, Beijing Forestry University, Beijing, China; ^3^College of Life Sciences, Hebei Agricultural University, Baoding, China

**Keywords:** VA-TIRFM, nanodomain, dynamics, biotic and abiotic stresses, flotillin-1, hypersensitive induced reaction-1

## Abstract

Plasma membranes are heterogeneous and contain multiple functional nanodomains. Although several signaling proteins have been shown to function by moving into or out of nanodomains, little is known regarding the effects of environmental cues on nanodomain organization. In this study, we investigated the heterogeneity and organization of distinct nanodomains, including those containing *Arabidopsis thaliana* flotillin-1 (AtFlot1) and hypersensitive induced reaction-1 proteins (AtHIR1), in response to biotic and abiotic stress. Variable-angle total internal reflection fluorescence microscopy coupled with single-particle tracking (SPT) revealed that AtFlot1 and AtHIR1 exhibit different lateral dynamics and inhabit different types of nanodomains. Furthermore, via SPT and fluorescence correlation spectroscopy, we observed lower density and intensity of AtFlot1 fluorescence in the plasma membrane after biotic stress. In contrast, the density and intensity of signal indicating AtHIR1 markedly increased in response to biotic stress. In response to abiotic stress, the density and intensity of both AtFlot1 and AtHIR1 signals decreased significantly. Importantly, SPT coupled with fluorescence recovery after photobleaching revealed that biotic and abiotic stress can regulate the dynamics of AtFlot1; however, only the abiotic stress can regulate AtHIR1 dynamics. Taken together, these findings suggest that a plethora of highly distinct nanodomains coexist in the plasma membrane (PM) and that different nanodomains may perform distinct functions in response to biotic and abiotic stresses. These phenomena may be explained by the spatial clustering of plasma membrane proteins with their associated signaling components within dedicated PM nanodomains.

## Introduction

The plasma membrane (PM) of eukaryotes delineates the interface between the cell and the environment. It plays a crucial role in transport processes, cell protection, endocytosis, and, principally, cell signaling (Morel et al., 2006; Grosjean et al., 2018). Multiple studies have shown that the PM is a highly heterogeneous organelle that can be subdivided into domains of distinct compositions, structures, and functions (Vieira et al., 2010). These domains are referred to as lipid rafts, membrane rafts, or micro- or nanodomains (Malinsky et al., 2013). They are enriched in certain lipids (cholesterol and sphingolipids) and were once thought to be identical to detergent-resistant membranes (DRMs) or detergent-insoluble membranes (DIMs) (Tanner et al., 2011). Lipid rafts have transient molecular associations with both lipid and protein components of the PM, providing a dynamic patchiness and local order in the fluid mosaic membrane (Mattson, 2005).

The microdomain concept has been widely confirmed by experimental evidence (e.g., superresolution microscopy, lipid analysis, and PM isolation) (Diaz-Rohrer et al., 2014; Shen et al., 2020). Recent observations of microdomains in live cells have provided a great deal of information regarding their dynamics. The dynamic behaviors of microdomain marker proteins, including hypersensitive induced reaction (HIR) proteins and flotillins, are dependent on the subcellular environment (Lv et al., 2017; Yu et al., 2017) and are closely related to protein biological functions. Therefore, dynamic analysis of microdomain marker proteins can reveal the underlying mechanisms of cell signal transduction (Yu et al., 2020a).

Hypersensitive induced reactions and flotillins form subfamilies within the stomatin/prohibitin/flotillin/HflK/C (SPFH) domain-containing family of proteins (Danek et al., 2020). They are highly mobile and heterogeneously distributed at the PM and are required for signal transmission (Haney and Long, 2010; Qi et al., 2011). Unlike other SPFH domain proteins, however, HIRs and flotillins from *Arabidopsis thaliana* (i.e., AtHIRs and AtFlots) tend to lack a transmembrane domain, making them peripheral membrane proteins (Danek et al., 2016). Using variable-angle total internal reflection fluorescence microscopy (VA-TIRFM) and structured illumination microscopy (SIM), Li et al. (2012) determined that flotillin-1 (Flot1) is heterogeneous and highly dynamic in the membranes of Arabidopsis epidermal cells. The dynamic behavior of Flot1 was also found to change significantly following elicitor flg22 treatment (Yu et al., 2017). Previous studies have shown that the endocytosis of Arabidopsis FLS2 and NRT1.1 is partially associated with the Flot1-associated endocytic pathway (Cui et al., 2018a; Zhang et al., 2019).

Unlike flotillins, HIRs are predominantly localized at the PM (Li et al., 2019). Using one-dimensional blue native (BN)-PAGE separation, Lv et al. (2017) demonstrated that levels of the AtHIR1 complex increase significantly following flg22 treatment. In addition, AtHIR1 dynamics markedly increase after treatment with methyl-β-cyclodextrins, which deplete cholesterol and disturb microdomains. In effect, it is clear that microdomains provide a platform for the efficient execution of highly specific signaling events.

Although HIRs and flotillins are involved in many biological processes, the molecular basis of their functions remains unclear. In this study, we combined single particle tracking (SPT), fluorescence correlation spectroscopy (FCS), and fluorescence recovery after photobleaching (FRAP) to measure the distribution and diffusion in the PM of AtFlot1 and AtHIR1. We also investigated the effects of biotic and abiotic stress on AtFlot1 and AtHIR1 density at the PM. We find that biotic and abiotic stress affect the dynamics of AtFlot1 and AtHIR1 to varying extents. The results of this work may provide useful insights into the molecular mechanisms of signaling by AtFlot1 and AtHIR1, as the spatiotemporal dynamics of nanodomains clearly play a role in signaling in responses to different stimuli and as different nanodomains may perform distinct functions.

## Results

### Nanodomains Exhibit Distinct Dynamics in Different Tissues

**FIGURE 1:**
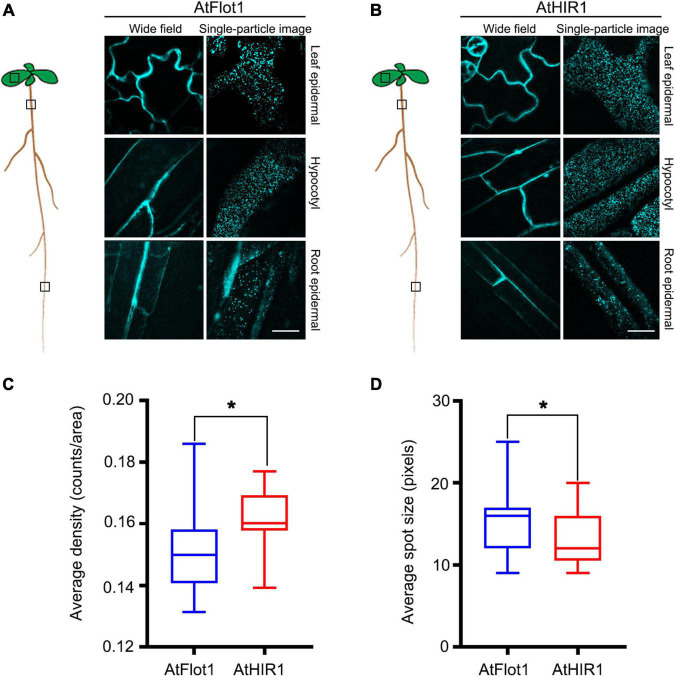
AtFlot1 and AtHIR1 label a variety of distinct membrane domains in epidermal cells of Arabidopsis. **(A,B)** Upper planes of leaf epidermal cells (top panels), elongating hypocotyl cells (middle panels), and root epidermal cells (bottom panels) were imaged in 5-d-old seedlings of Arabidopsis plants expressing GFP-AtFlot1 **(A)** and AtHIR1-GFP **(B)**. Bar = 5 μm. **(C)** Average fluorescence density of GFP-AtFlot1 and AtHIR1-GFP fluorescent spots. For each examination, at least 50 cells from each of five seedlings were examined. In each case, three biological replicates were performed. Statistical significance was determined using Student’s *t*-tests (*P < 0.05). **(D)** Average size of GFP-AtFlot1 and AtHIR1-GFP fluorescent spots. For each examination, at least 30 cells from each of five seedlings were examined. In each case, three biological replicates were performed. Statistical significance was determined using Student’s *t*-tests (*P < 0.05).

To gain insight into the behavior of the AtFlot1 and AtHIR1 *in vivo*, we used VA-TIRFM to monitor the dynamics of individual AtFlot1 and AtHIR1 particles at the PM with high resolution in the epidermal cells of different tissues. For both proteins, we observed distinct spots with almost constant fluorescence; in addition, patchy localization patterns were observed in leaf and elongating hypocotyl epidermal cells (Figures 1A,B). In addition, we found that the moving AtFlot1 and AtHIR1 particles appeared as well-dispersed diffraction-limited fluorescent spots that displayed high-speed or low-speed dynamics at the PM. Moreover, some GFP-AtFlot1-associated spots disappeared from the PM after a long period of residence, whereas other spots appeared to rise up from within the cell. On the other hand, most of the AtHIR1-GFP fluorescent spots stayed at the PM during the entire observation period. By contrast, neither protein exhibited a fluorescence pattern consistent with localization to distinct membrane domains in root epidermal cells (Figures 1A,B, bottom panels). Together, these results indicate that AtFlot1 and AtHIR1 are distributed differently in different tissues.

We next quantified the densities and sizes of AtFlot1- and AtHIR1-containing particles. The AtFlot1 particle density, expressed as mean value ± SD, was 0.15 ± 0.014 counts/area in the Arabidopsis leaf, while the AtHIR1 particle density in the same tissue was 0.16 ± 0.009 counts/area (Figure 1C). The density increased slightly in Arabidopsis, but the difference was statistically significant. The mean size of AtFlot1-containing particles in leaf tissues was 3.95 × 3.95 ± 0.59 pixels, whereas the mean size of AtHIR1 particles was 3.62 × 3.62 ± 0.48 pixels (Figure 1D). Thus, AtFlot1 and AtHIR1 exist in different types of nanodomains within the PM.

### The Dynamic Behavior of AtFlot1 and AtHIR1 in the Plasma Membrane

**FIGURE 2:**
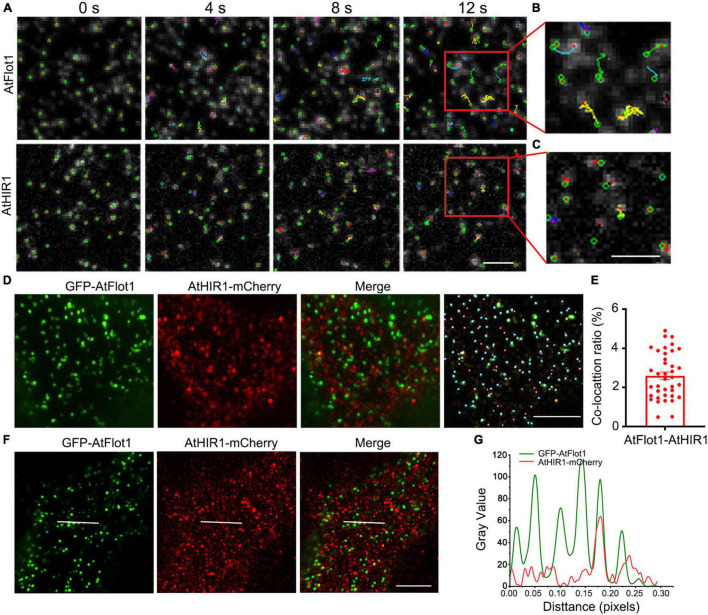
TIRFM analysis of the association of AtFlot1 and AtHIR1 proteins. **(A)** Time-lapse trajectories of AtFlot1/AtHIR1 foci shown at different time points (0, 4, 8, and 12 s). The foci moved over a significantly wider area in AtFlot1 plants, and AtHIR1 foci showed smaller movements. Bar = 1 μm. **(B,C)** The trajectory of fluorophore-tagged molecules from the area indicated by the red box in **(A)**, showing varied trajectories among different spots. Bar = 1 μm. **(D)** VA-TIRFM images of leaf epidermal cells co-expressing GFP-AtFlot1 and AtHIR1-mCherry. The cyan ball represents GFP-AtFlot1 and the carmine balls represent AtHIR1-mCherry. Bars = 5 μm. **(E)** Histogram showing the ratios of co-localization between GFP-AtFlot1 and AtHIR1-mCherry. For each examination, at least 30 cells from each of five seedlings were examined. In each case, three biological replicates were performed. **(F)** Colocalization of GFP-AtFlot1 foci with AtHIR1-mCherry foci. The white line indicates fluorescence signals. Bar = 5 μm. **(G)** GFP-AtFlot1 and AtHIR1-mCherry fluorescence signals for the events shown in **(F)**. The green line indicates GFP-AtFlot1, and the red line indicates AtHIR1-mCherry.

Our result showed that the sizes and densities of particles containing the two proteins were different, and individual AtFlot1 and AtHIR1 particles appeared at the membrane as isolated fluorescent spots and demonstrated dramatically different dynamics. Therefore, in order to further evaluate the dynamics of AtFlot1 and AtHIR1 particles at the PM, we used SPT to investigate particle trajectories. We found that AtFlot1-associated particles have a wider range of motion than do AtHIR1-associated particles; only a few AtFlot1-labeled foci displayed motion patterns similar to those of AtHIR1 particles (Figures 2A–C).

We generated transgenic Arabidopsis coexpressing GFP-AtFlot1 and AtHIR1-mCherry. VA-TIRFM, coupled with the use of Imaris image analysis software (Bitplane), revealed that AtFlot1 and AtHIR1 signals showed only a 2.25 ± 0.9% overlap (*n* = 39 images from five seedlings) (Figures 2D,E). Furthermore, we also analyzed the fluorescence signals of AtFlot1 and AtHIR1 on the surfaces of cells. Here, the results showed that AtFlot1 foci displayed a higher fluorescence intensity than did AtHIR1 foci. More importantly, the fluorescent signals of GFP-AtFlot1 showed little co-localization with those of AtHIR1-mCherry (Figures 2F,G).

**FIGURE 3:**
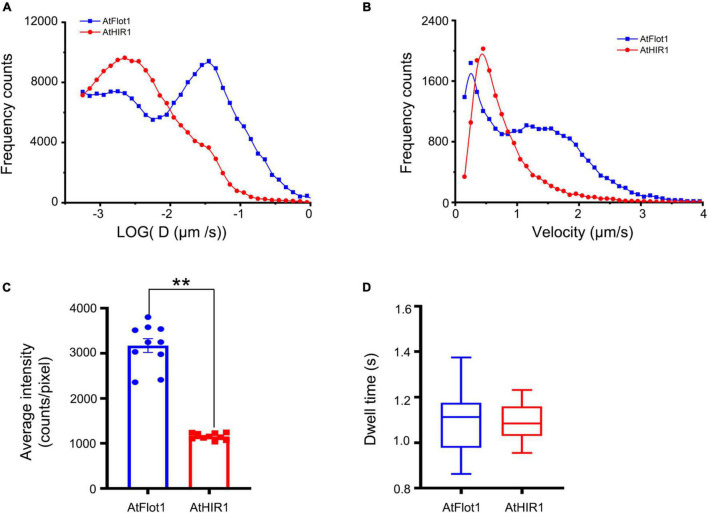
Assessing AtFlot1 and AtHIR1 protein mobility by TIRFM analysis. **(A)** Distribution of the diffusion coefficients of AtFlot1 (blue, *n* = 1892) and AtHIR1 (red, *n* = 2513) in living leaf epidermal cells. **(B)** Distribution of the velocity of AtFlot1 (blue, *n* = 2243) and AtHIR1 (red, *n* = 1954) in living leaf epidermal cells. **(C)** Average fluorescence intensity of GFP-AtFlot1 and AtHIR1-GFP. For each examination, at least 10 cells from each of five seedlings were examined. In each case, three biological replicates were performed. Statistical significance was determined using Student’s *t*-tests (**P < 0.01). **(D)** Dwell times were analyzed in GFP-AtFlot1 and AtHIR1-GFP plants. For each examination, at least 30 cells from each of five seedlings were examined. In each case, three biological replicates were performed. Statistical significance was determined using Student’s *t*-tests.

We further measured the dynamic properties of AtFlot1 and AtHIR1 particles by calculating their diffusion coefficients and velocities. We calculated the diffusion coefficients by linear fitting of mean square displacement vs. time (MSD-t) and plotted these diffusion coefficients in histograms with logarithmically spaced bins. We considered the position of the peak (Ĝ) to be the characteristic diffusion coefficient. We found that diffusion coefficients of AtFlot1 could be distributed into two subpopulations: a small Ĝ, 1.8 × 10^–3^ μm^2^/s, and a large Ĝ, 3.5 × 10^–2^ μm^2^/s. In contrast, the Ĝ of AtHIR1 particles was distributed into one subpopulation of 2.2 × 10^–3^ μm^2^/s (Figure 3A).

Upon calculating the velocities of the signals of AtFlot1 and AtHIR1, we found that AtFlot1 exhibited a bimodal distribution of velocities and that AtHIR1 exhibited a unimodal distribution. The Ĝ values of AtFlot1 were 0.25 and 1.15 μm/s, respectively, and the Ĝ of AtHIR1 was 0.45 μm/s (Figure 3B). AtFlot1 was confined to a relatively larger area than was AtHIR1.

We compared the intensities of GFP-AtFlot1 and AtHIR1-GFP particles as shown in Figure 3C. Quantitative image analyses of the TIRFM images indicated that the GFP-AtFlot1 particle fluorescence intensity, expressed as mean ± SD, was 3170.14 ± 459.86 counts/pixel, whereas the fluorescence intensity of AtHIR1-GFP particles was only 1156.33 ± 68.64 counts/pixel (Figure 3C and Supplementary Figure 1). While it should be noted that minor fluorescence intensity variations may appear because of slight differences in the angle of VA-TIRFM between measurements, the clear significant differences that we identified are not likely to be explained in this way. However, to further investigate the underlying differences, we further analyzed the lifetimes of GFP-AtFlot1 and AtHIR1-GFP particles, in terms of the dwell time (τ value), which is defined as the duration in which a protein remains at the cell surface prior to endocytosis (Flores-Otero et al., 2014). When the frequency distribution was fitted with an exponential function, the τ value was 1.11 ± 0.13 s in cells expressing GFP-AtFlot1 (Figure 3D), which was not significantly different than the τ value of AtHIR1-GFP particles, 1.09 ± 0.07 s (Figure 3D). These results suggest that both AtFlot1 and AtHIR1 form particles that are non-randomly distributed across the PM. Thus, the two proteins were comparable in dynamics, intensity, and dwell time despite their significant differences in dynamic features.

### AtFlot1 and AtHIR1 Distribution Are Controlled by Biotic and Abiotic Stresses

The distributions of fluorescence intensities of nanodomains are typically affected by multiple stimuli, implying that nanodomains may play a role in signal transduction. We investigated whether the fluorescence intensity distributions of AtFlot1 and AtHIR1 are associated with biotic and abiotic stresses. We first mimicked biotic stress by the application of exogenous peptides, including the conserved 22-amino-acid epitope flagellin 22 (flg22), which is recognized by most land plants through the leucine-rich repeat receptor kinase FLS2 (Li et al., 2016; Zipfel and Oldroyd, 2017), and a 23-amino acid endogenous peptide Pep1, which triggers defenses through the leucine-rich repeat receptor kinase PEPR1 (Zhou and Yang, 2016; Zhou and Zhang, 2020).

**FIGURE 4:**
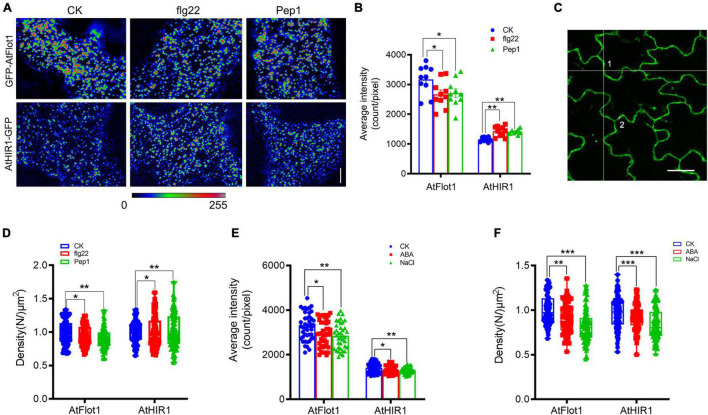
Distribution of GFP-AtFlot1 and AtHIR1-GFP at the PM under biotic and abiotic stresses. **(A)** Pseudocolor images (blue-yellow-red palette) show the fluorescence intensity of AtFlot1/AtHIR1 foci at the plasma membrane in control cells and cells treated with flg22 or Pep1 peptides for 15 min. Bar = 5 μm. **(B)** Average fluorescence intensity of GFP-AtFlot1 and AtHIR1-GFP under control, flg22 and Pep1 treatments for 15 min. For each examination, at least 10 cells from each of three seedlings were examined. In each case, three biological replicates were performed. Statistical significance was determined using Student’s *t*-tests (*P < 0.05; **P < 0.01). **(C)** Confocal images of GFP-AtFlot1 were analyzed using FCS at points 1 and 2. Bar = 10 μm. **(D)** The density of GFP-AtFlot1 and AtHIR1-GFP under flg22 and Pep1 treatment was measured by FCS. For each examination, at least 30 cells from each of five seedlings were examined. In each case, three biological replicates were performed. Statistical significance was determined using Student’s *t*-tests (*P < 0.05; **P < 0.01). **(E)** Average fluorescence intensity of GFP-AtFlot1 and AtHIR1-GFP under control and ABA and NaCl treatment conditions each for 15 and 10 min. For each examination, at least 30 cells from each of five seedlings were examined. In each case, three biological replicates were performed. Statistical significance was determined using Student’s *t*-tests (*P < 0.05; **P < 0.01). **(F)** The density of GFP-AtFlot1 and AtHIR1-GFP upon treatment with ABA and NaCl was measured by FCS. For each examination, at least 30 cells from each of five seedlings were examined. In each case, three biological replicates were performed. Statistical significance was determined using Student’s *t*-tests (**P < 0.01 and ***P < 0.001).

We analyzed the fluorescence intensity distribution of AtFlot1 and AtHIR1 spots before and after peptide treatment. Under conditions that reflected the steady state configuration prior to pathogen attack, AtFlot1 and AtHIR1 foci at the PM showed heterogeneous fluorescence intensities (Figure 4A) with average values of 3170.1 ± 459.9 counts/pixel and 1162.4 ± 68.6 counts/pixel, respectively (Figure 4B). After the seedlings of AtFlot1 were treated with flg22 or Pep1 for 15 min, the fluorescence intensities of most AtFlot1 foci markedly decreased to average values of 2672.3 ± 426.5 counts/pixel for flg22 and 2708.9 ± 426.2 counts/pixel for Pep1 (Figure 4B and Supplementary Figure 1). By contrast, flg22 or Pep1 treatment significantly increased the fluorescence intensity of AtHIR1 foci to average values of 1652.99 ± 14.97 counts/pixel for flg22 and 1412.3 ± 79.3 counts/pixel for Pep1 (Figure 4B and Supplementary Figure 1).

**FIGURE 5:**
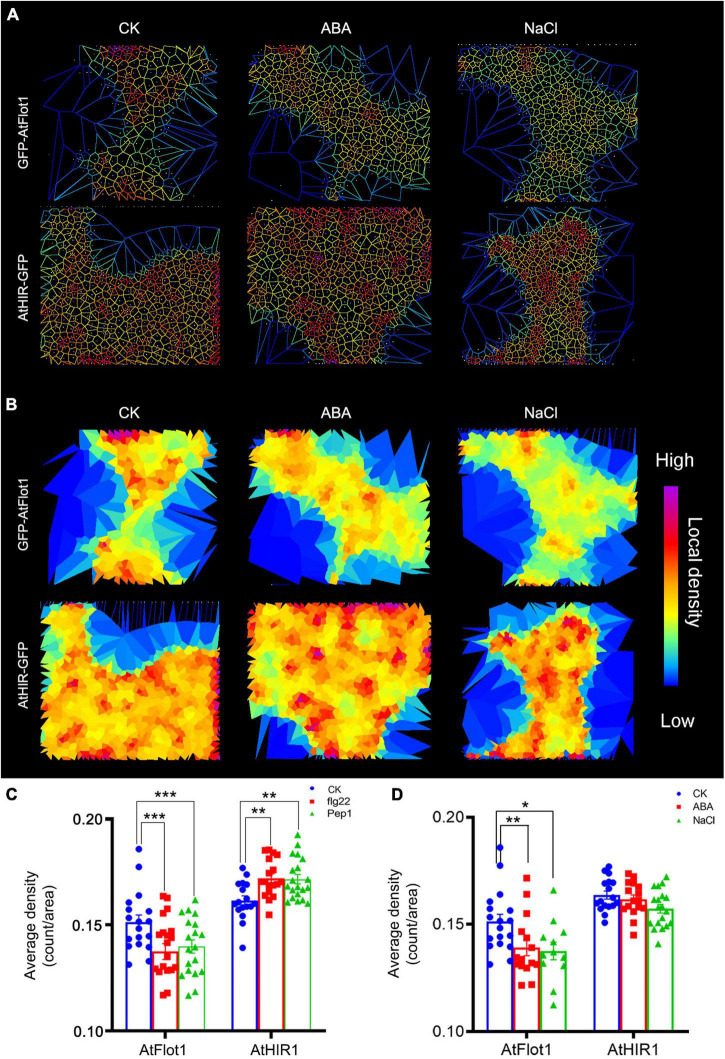
Density of GFP-AtFlot1 and AtHIR1-GFP at the PM under biotic and abiotic stresses. **(A,B)** Cell identification and segmentation in a multicellular raw image. Recognition and segmentation of cells in PM by Imaris software based on protein density and Voronoï tessellation setting of the threshold of localization density. **(C)** The fluorescence density of GFP-AtFlot1 and AtHIR1-GFP at the PM in leaf epidermal cells under control and flg22 and Pep1 treatment conditions. For each examination, at least 20 cells from each of five seedlings were examined. In each case, three biological replicates were performed. Statistical significance was determined using Student’s *t*-tests (**P < 0.01 and ***P < 0.001). **(D)** The fluorescence density of GFP-AtFlot1 and AtHIR1-GFP at the PM in leaf epidermal cells treated with ABA and NaCl. For each examination, at least 30 cells from each of five seedlings were examined. In each case, three biological replicates were performed. Statistical significance was determined using Student’s *t*-tests (*P < 0.05; **P < 0.01).

We applied FCS to precisely calculate the densities of AtFlot1 and AtHIR1 within different plant immune system environments as well. FCS measurement was established by focusing an excitation laser beam onto the membrane and then monitoring fluorescence fluctuations within the focal volume of the laser beam (Li et al., 2016; Zhu et al., 2020). As shown in Figures 4C,D, flg22 and Pep1 treatment decreased AtFlot1 density as per FCS. By contrast, the abundance of AtHIR1 molecules at the plasma membrane further increased after treatment with flg22 and Pep1 (Figure 4D). Similar results were found in a parallel experiment using SPT (Figure 5C).

In addition to biotic stress, plants and animals can perceive extracellular abiotic signals by cell surface receptors (Bucherl et al., 2017). To investigate whether abiotic stress affects AtFlot1 and AtHIR1 distributions, we treated Arabidopsis with abscisic acid (ABA) and NaCl, which are commonly used to simulate drought conditions. It is now well accepted that ABA plays important roles in plant adaptation to environmental stresses including drought or high salinity (Gong et al., 2020). In cells treated with ABA or NaCl, the fluorescence intensities of GFP-AtFlot1 and AtHIR1-GFP significantly decreased compared with the control (AtFlot1: 3262 ± 595.3 counts/pixel for control, 2940.2 ± 588.3 counts/pixel for ABA and 2847.4 ± 565.3 counts/pixel for NaCl; AtHIR1: 1407.8 ± 223.3 counts/pixel for control, 1323 ± 181.4 counts/pixel for ABA and 1296.4 ± 137.8 counts/pixel for NaCl) (Figure 4E and Supplementary Figures 2, 3). The fluorescence intensities of GFP-AtFlot1 and AtHIR1-GFP significantly decreased as well (AtFlot1: 8.8% for ABA and 8% NaCl; AtHIR1: 17.4% for ABA and 15.6% NaCl) (Figure 4F).

To further analyze these densities, we used Imaris image analysis software to segment the cells. The results showed that treatment with ABA or NaCl significantly changed the densities of AtFlot1, but the densities of AtHIR1 barely changed (Figures 5A,B). Similar results were observed using SPT (Figure 5D). These data indicate that membrane nanodomains have dynamic distributions under different environmental conditions.

### Biotic Stress Affects AtFlot1 and AtHIR1 Nanodomain Dynamics

Previous studies have shown that nanodomains are associated with plant-microbe interactions (El Yahyaoui et al., 2004; Cui et al., 2018a). Accordingly, we tested whether the presence of exogenous peptides influences the dynamic behavior of AtFlot1 and AtHIR1. We compared the lateral mobilities of AtFlot1 and AtHIR1 particles under different conditions.

**FIGURE 6:**
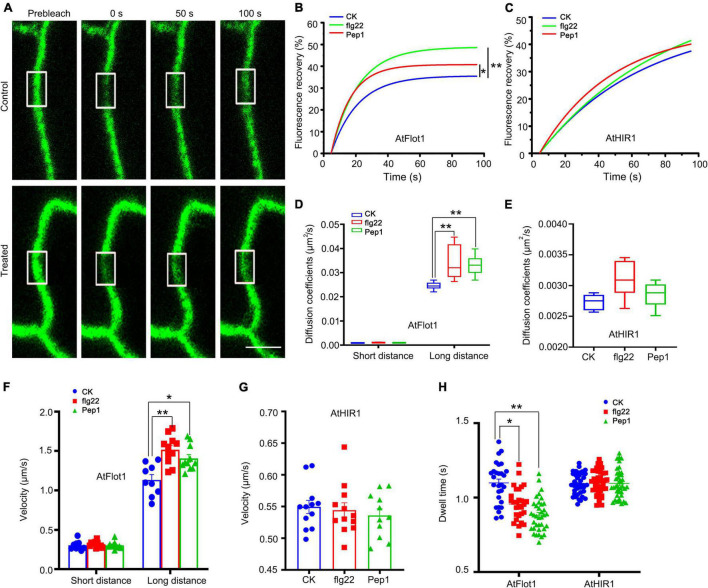
Dynamics of GFP-AtFlot1 and AtHIR1-GFP at the PM under control and flg22 and Pep1 treatment conditions. **(A)** FRAP time course of cells expressing GFP-AtFlot1. White squares indicate bleached regions. Bar = 5 μm. **(B)** Fluorescence recovery curves of the photobleached region of interest in GFP-AtFlot1-expressing cells under control and flg22 and Pep1 treatment conditions. For each examination, at least 15 cells from each of five seedlings were examined. In each case, three biological replicates were performed. Statistical significance was determined using Student’s *t*-tests (*P < 0.05; **P < 0.01). **(C)** Fluorescence recovery curves of the photobleached region of interest in AtHIR1-GFP-expressing cells under control and flg22 and Pep1 treatment conditions. For each examination, at least 15 cells from each of five seedlings were examined. In each case, three biological replicates were performed. Statistical significance was determined using Student’s *t*-tests. **(D)** Long-distance and short-distance diffusion coefficients for GFP-AtFlot1 in different environments. For each examination, at least 30 cells from each of five seedlings were examined. In each case, three biological replicates were performed. Statistical significance was determined using Student’s *t*-tests (**P < 0.01). **(E)** Diffusion coefficients of AtHIR1-GFP under different conditions. For each examination, at least 30 cells from each of five seedlings were examined. In each case, three biological replicates were performed. Statistical significance was determined using Student’s *t*-tests. **(F)** The velocity of GFP-AtFlot1 under different conditions. For each examination, at least 15 cells from each of five seedlings were examined. In each case, three biological replicates were performed. Statistical significance was determined using Student’s *t*-tests (*P < 0.05; **P < 0.01). **(G)** The velocity of AtHIR1-GFP under different conditions. For each examination, at least 15 cells from each of five seedlings were examined. In each case, three biological replicates were performed. Statistical significance was determined using Student’s *t*-tests. **(H)** Dwell times of GFP-AtFlot1 and AtHIR-GFP under control and flg22 and Pep1 treatment conditions. For each examination, at least 20 cells from each of five seedlings were examined. In each case, three biological replicates were performed. Statistical significance was determined using Student’s *t*-tests (*P < 0.05; **P < 0.01).

First, FRAP assays were performed to examine the dynamics of AtFlot1 and AtHIR1. The initial bleaching region was divided into three parts, designated (op, middle, and bottom) (Supplementary Figure 4A). These FRAP experiments showed that for AtFlot1 and AtHIR1, recovery of fluorescence in the middle of the bleached area was slower than that in the periphery (Supplementary Figure 4). This suggests that AtFlot1 and AtHIR1 undergo lateral diffusion in the membrane. In addition, further FRAP analysis showed that in cells treated with flg22 or Pep1, AtFlot1 the percentage of recovery increased relative to untreated cells (control, 35.5%; flg22 treated, 48.6%; Pep1 treated, 40.7%) (Figures 6A,B). By contrast, in the AtHIR1-GFP cell lines, kinetic profiles were similar before and after flg22 or Pep1 treatment (Figure 6C).

We used TIRFM to further verify the effects of flg22 and Pep1 on the lateral diffusion of AtFlot1 and AtHIR1. The diffusion coefficient and velocity of AtFlot1 particles were divisible into two subpopulations (Supplementary Figure 5). There were small, similar Ĝ values of diffusion coefficients for AtFlot1, but there were also large Ĝ values of diffusion coefficients at 2.46 × 10^–2^ μm^2^/s (SE: 2.31–2.61 × 10^–2^ μm^2^/s) in the control seedlings (Figure 6D and Supplementary Figure 5). After treatment with flg22 or Pep1, the large Ĝ of diffusion coefficients was 3.41 × 10^–2^ μm^2^/s (SE: 2.76–4.06 × 10^–2^ μm^2^/s) and 3.31 × 10^–2^ μm^2^/s (SE: 2.92–3.70 × 10^–2^ μm^2^/s), respectively (Figure 6D and Supplementary Figure 5), indicating that the diffusion coefficients of AtFlot1 increased in the presence of flg22 or Pep1. We also found that the Ĝ of velocity was 0.31 ± 0.067 μm/s and 1.20 ± 0.18 μm/s in control seedlings (Figure 6F and Supplementary Figure 6). After flg22 or Pep1 treatment, the large Ĝ of velocity was 1.52 ± 0.17 μm/s and 1.41 ± 0.15 μm/s, respectively, marking a significant increase over the control seedlings (Figure 6F and Supplementary Figure 6).

We found that the diffusion coefficients and velocity of AtHIR1 were distributed into a single population (Supplementary Figure 5). The diffusion coefficient and velocity appeared to change slightly in response to flg22 or Pep1 treatment, but these changes were not significant (diffusion coefficient: control: 2.73 × 10^–3^ μm^2^/s, SE: 2.61–2.85 × 10^–3^μm^2^/s; flg22: 3.07 × 10^–3^μm^2^/s, SE: 2.80–3.34 × 10^–3^μm^2^/s; Pep1: 2.85 × 10^–3^μm^2^/s, SE: 2.65–3.05 × 10^–3^μm^2^/s; Velocity: control: 0.55 ± 0.034 μm/s; flg22: 0.54 ± 0.039 μm/s; Pep1: 0.54 ± 0.033 μm/s) (Figures 6E,G and Supplementary Figures 5, 6). These data indicate that the lateral movements of AtFlot1 and AtHIR1 particles changed to varying extents in response to flg22 or Pep1.

In addition to lateral diffusion, some vertical movement was noted, as well. Specifically, while some fluorescent spots remained at the cell surface, others gradually moved out of the focal plane. We analyzed the dwell time of AtFlot1 and AtHIR1 particles, accordingly. The τ values of GFP-AtFlot1 under flg22 treatment (0.96 ± 0.11 s) and under Pep1 treatment (0.84 ± 0.13 s) were significantly lower than the τ value of AtFlot1 particles under control conditions (1.11 ± 0.13 s; Figure 6H). In contrast, the τ value of AtHIR1 was not significantly affected by flg22 or Pep1 treatment (control: 1.09 ± 0.07 s; flg22: 1.10 ± 0.10 s; Pep1: 1.11 ± 0.11 s; Figure 6H). Thus, the dwell time of AtFlot1-containing particles on the PM was shorter than that of AtHIR1-containing particles, and the dwell time of AtFlot1-containing particles was uniquely responsive to flg22 and Pep1 treatment. These results suggest that AtFlot1 and AtHIR1 participate in plant immunity via different dynamic behaviors.

### Abiotic Stress Regulates AtFlot1 and AtHIR1 Nanodomain Dynamics

**FIGURE 7:**
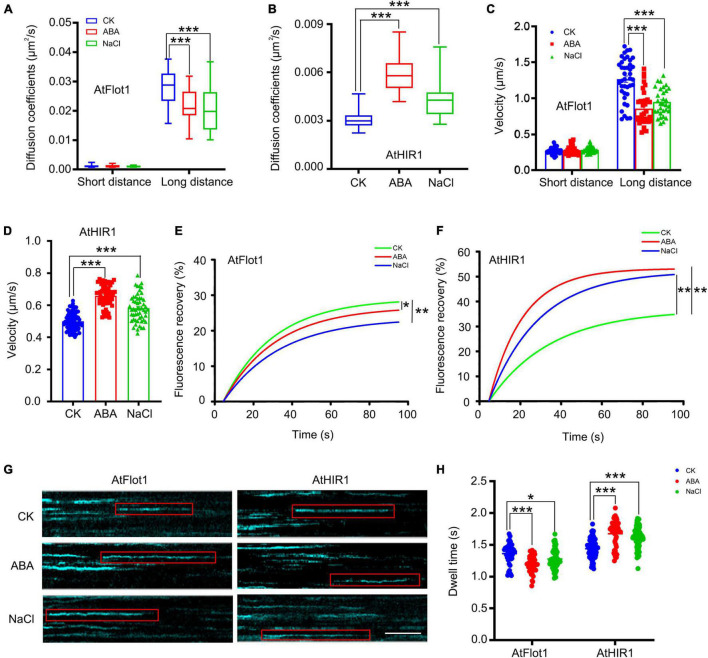
Dynamics of GFP-AtFlot1 and AtHIR1-GFP at the PM under control and ABA and NaCl treatment conditions. **(A)** Long-distance and short-distance diffusion coefficients for GFP-AtFlot1 under different conditions. For each examination, at least 30 cells from each of five seedlings were examined. In each case, three biological replicates were performed. Statistical significance was determined using Student’s *t*-tests (***P < 0.001). **(B)** Diffusion coefficients of AtHIR1-GFP in different environments. For each examination, at least 30 cells from each of five seedlings were examined. In each case, three biological replicates were performed. Statistical significance was determined using Student’s *t*-tests (***P < 0.001). **(C)** The velocity of GFP-AtFlot1 under different conditions. For each examination, at least 30 cells from each of five seedlings were examined. In each case, three biological replicates were performed. Statistical significance was determined using Student’s *t*-tests (***P < 0.001). **(D)** The velocity of AtHIR1-GFP under different conditions. For each examination, at least 30 cells from each of five seedlings were examined. In each case, three biological replicates were performed. Statistical significance was determined using Student’s *t*-tests (***P < 0.001). **(E)** Fluorescence recovery curves of the photobleached region of interest in GFP-AtFlot1 cells under control and ABA and NaCl treatment conditions. For each examination, at least 15 cells from each of five seedlings were examined. In each case, three biological replicates were performed. Statistical significance was determined using Student’s *t*-tests (*P < 0.05; **P < 0.01). **(F)** Fluorescence recovery curves of the photobleached region of interest in AtHIR1-GFP cells under control and ABA and NaCl treatment conditions. For each examination, at least 15 cells from each of five seedlings were examined. In each case, three biological replicates were performed. Statistical significance was determined using Student’s *t*-tests (**P < 0.01). **(G)** Representative kymographs showing individual GFP-AtFlot1 and AtHIR1-GFP dwell times in the presence of the control, ABA, and NaCl treatments. Bar = 2 s. **(H)** Dwell times of GFP-AtFlot1 and AtHIR-GFP treated with control, ABA, and NaCl. For each examination, at least 30 cells from each of five seedlings were examined. In each case, three biological replicates were performed. Statistical significance was determined using Student’s *t*-tests (*P < 0.05, ***P < 0.001).

Nanodomain marker proteins play crucial roles in plant resistance to abiotic stress (Checker and Khurana, 2013). To investigate whether abiotic stress affects nanodomain marker protein diffusion, we observed the dynamics of GFP-AtFlot1 and AtHIR1-GFP in response to ABA and NaCl treatment. When seedlings were treated with ABA, the large Ĝ of the diffusion coefficient of GFP-AtFlot1 particles was 2.18 × 10^–2^ μm^2^/s (SE: 1.58–2.78 × 10^–2^ μm^2^/s) (Figure 7A and Supplementary Figure 7) and that of velocity was 0.86 ± 0.23 μm/s (Figure 7C and Supplementary Figure 8), indicating that the dynamics of AtFlot1 significantly decreased. Unexpectedly, in AtHIR1-GFP Arabidopsis plants, the diffusion coefficient and velocity values of AtHIR1-GFP significantly increased in response to ABA treatment (Figures 7B,D).

We also analyzed GFP-AtFlot1 and AtHIR1-GFP dynamics in seedlings treated with NaCl. We found that the large Ĝ of diffusion coefficient (2.08 × 10^–2^ μm^2^/s; SE: 1.38–2.78 × 10^–2^ μm^2^/s) and large Ĝ of velocity (0.95 ± 0.019 μm/s) of AtFlot1 particles significantly decreased compared with the untreated control value (Figures 7A,C and Supplementary Figures 7, 8). By contrast, the diffusion coefficient (4.22 × 10^–3^ μm^2^/s; SE: 3.32–5.12 × 10^–3^ μm^2^/s) and velocity (0.58 ± 0.08 μm/s) of AtHIR1 significantly increased (Figures 7B,D). When we photobleached GFP signals on the cell surface and measured FRAP signals from AtFlot1 and AtHIR1 over time, we found that after ABA and NaCl treatment, the lateral diffusion of GFP-AtFlot1 and AtHIR1-GFP was markedly altered compared to that in normal lines. Thus, abiotic stress changed the dynamic behavior of AtFlot1 and AtHIR1 (Figures 7E,F). Abiotic stress appears to strongly affect the trajectory and speed of PM protein diffusion.

To further test whether abiotic stress plays a role in the dynamic behavior of AtFlot1 and AtHIR1 in Arabidopsis, we conducted a kymograph analysis based on space-time segmentation. This type of analysis more effectively exploits spatiotemporal information than common frame-by-frame tracking methods (Smal et al., 2010). As shown in Figure 7G, vertical lines in the kymographs represent the lateral stability of AtFlot1 and AtHIR1 particles. Upon ABA and NaCl treatment, the vertical lines became curved (Figure 7G).

We further compared the dwell times of GFP-AtFlot1 and AtHIR1-GFP before and after ABA and NaCl treatment. The τ value of GFP-AtFlot1 was 1.20 ± 0.14 s following ABA treatment and was 1.28 ± 0.13 s after NaCl treatment, indicating that these treatments significantly decreased dwell times compared to the control seedlings (control: 1.36 ± 0.16 s) (Figure 7H). ABA and NaCl treatments increased the dwell times of AtHIR1-GFP at the PM compared to the control seedlings (control: 1.44 ± 0.14 s; ABA: 1.68 ± 0.20 s; NaCl: 1.62 ± 0.16 s) (Figure 7H), suggesting that the dwell times of GFP-AtFlot1 and AtHIR1-GFP at the PM were heterogeneous under ABA and NaCl treatments. Taken together, our findings suggest that nanodomain dynamics are associated with abiotic stress signal transduction and that different nanodomains perform different functions in response to abiotic stress.

## Discussion

The PM consists of a mosaic of functional nanodomains that are responsible for a variety of physiological processes associated with the cell surface (Laude and Prior, 2004; Cui et al., 2019; Yu et al., 2020a). Studying membrane nanodomain dynamics in response to environmental changes in living cells may reveal possible early signal transduction processes, thereby elucidating other signal transduction networks in plants. However, little is known about the relationship between nanodomain dynamics and the distinct signaling outputs that occur as plants perceive environment changes. Here, we provide evidence that Atflot1 and AtHIR1 are heterogeneously distributed within the PM by the formation of distinct PM nanodomain localization patterns. We used parallel quantitative approaches with VA-TIRFM and FCS to directly observe the densities of AtFlot1 and AtHIR1 to identify any significant changes between them in response to different stimuli. After a variety of environmental stimuli, AtFlot1 and AtHIR1 showed significant differences in mobility in VA-TIRFM and FRAP analyses. We propose that the dynamic behaviors of nanodomain marker proteins are dependent on subcellular environments and are closely related to the biological functions of these proteins.

A previous study reported that various types of specialized nanodomain act as molecular scaffolds to mediate domain platform assembly, and further, that they may be involved in different biological processes (Jarsch et al., 2014). Our present work extends this previous work on nanodomains by not only examining the distribution and dynamics of AtFlot1 and AtHIR1 but also analyzing co-localization between these two proteins. Similarly, Hao et al. (2014) reported that RbohD clustering that occurred following recruitment to membrane nanodomains permitted the fine-tuning of signal transduction. Here, we not only examined the distribution and dynamics of AtFlot1 and AtHIR1 but also analyzed co-localization between AtFlot1 and AtHIR1. Our results showed that the trajectories, lateral diffusion characteristics, densities, spot sizes, intensities, and dwell times of AtFlot1 and AtHIR1 differ, implying that the nanodomain dynamics that occur in distinct biological circumstances are more detailed than previously suggested (Figures 2, 3). Specifically, our lateral diffusion analysis demonstrated that AtFlot1 moves faster than AtHIR1 (Figures 2, 3). Moreover, we found that the fluorescent signals of AtFlot1 showed little co-localization with AtHIR1, indicating their spatial separation within the PM. We propose that mechanisms governing the movement of these particles may account for the fact that different membrane nanodomain contain different types of membrane-resident proteins that perform different biological functions.

The spatial and temporal dynamics of membrane proteins provide important indicators of many fundamental cellular processes (Yu et al., 2020b); for instance, environmental stresses trigger lateral movements of membrane proteins (Cui et al., 2021). Several studies have also shown that the fluctuations in the densities of membrane proteins can reveal the dynamics of membranes (Li et al., 2016), and these changes in density have been found to be related to signaling pathway activity. For example, the density of FLS2 decreases in response to its ligand, flg22 (Cui et al., 2018a), and Xing et al. (2019) showed that chitin treatment induces an increase in the density of PLDδ-GFP at the PM *in vivo*, indicating that PLDδ was rapidly recruited from the cytoplasm to the PM by secretion following chitin treatment. Therefore, we also explored the relationship between signal transduction and the abundance of nanodomain proteins. We found that the intensity of fluorescent signals associated with AtFlot1 and the density of the protein at the PM markedly decreased following flg22 or Pep1 treatment, whereas these factors related to AtHIR1 significantly increased after flg22 or Pep1 treatment (Figures 4, 5). We conclude that flg22 and Pep1 enhance the internalization of AtFlot1, but they do not induce AtHIR1 endocytosis. However, in the presence of ABA or NaCl, the intensity and density of GFP-AtFlot1 signal at the PM markedly decreased compared with control cells (Figures 4, 5). These findings suggest that the organization and dynamics of nanodomains are affected by different stimuli, implying that different nanodomains may possess different biological function. In addition, the same stimuli showed different dynamic on different nanodomains protein. We suspect that the structure of the protein may be responsible for different dynamic and function. An increasing number of studies have demonstrated that AtHIRs have palmitoylated (Hemsley et al., 2013) or myristoylated (Majeran et al., 2018), however, AtFlots have no myristoylation or palmitoylation motif. AtFlots presence a putative membrane binding site in N-termini, but the site was not detected in AtHIRs (Danek et al., 2020).

Mounting evidence indicates that the mobility of proteins at the PM is a critical determinant of protein-protein interactions, which control complex cellular processes (Owen et al., 2009). Similarly, biophysical and microscopy analyses have shown that the dynamic behaviors of proteins, which are dependent on the subcellular environment, are closely related to protein biological functions (Yu et al., 2020a). For instance, Bucherl et al. (2017) monitored the lateral mobility of FLS2 and BRI1 at the PM and found that ligand binding can affect the lateral mobility of both receptors, suggesting that the lateral movements of membrane proteins may play key roles in cellular responses to environmental stress. Therefore, the spatial and temporal dynamic analysis of proteins can reveal information regarding the underlying mechanisms of cell signal transduction. We used VA-TIRFM combined with FRAP to observe the complex kinetics at the single-molecule level in living plant cells (Li et al., 2016; Cui et al., 2018b). Our result showed that the lateral mobility of AtFlot1 significantly increased in the presence of flg22 or Pep1 (Figures 6B,D,F), but was not affected in AtHIR1 (Figures 6C,E,G). In addition, flg22 and Pep1 induced contrasting changes in the dwell time of AtFlot1 vs. AtHIR1 (Figure 6H). Based on the enhancement of the endocytosis of AtFlot1 under biotic stress, we speculate that the biotic stress might trigger innate immune responses in plants by regulating the dynamic endocytosis of certain proteins.

Plants have developed specific mechanisms that allow them to rapidly perceive and respond to stress in their environment (Danquah et al., 2014; Li et al., 2016). Abiotic stress is a severe environmental element that impairs productivity in crop systems (Hirayama and Shinozaki, 2010; Danquah et al., 2014). The signaling pathway leading to response to ABA or NaCl has been identified as a central regulator of abiotic stress responses in plants, triggering major changes in gene expressions and adaptive physiological responses (Hao et al., 2014; Su et al., 2021). Despite an abundance of plant protein dynamics, which important in many fundamental biological processes (Zhang et al., 2009), nanodomain marker protein dynamics in response to abiotic stress have not been reported to date. Therefore, we focused on the dynamics of nanodomain marker proteins in plant resistance to abiotic stress. We found that simulated abiotic stress decreased the velocity and diffusion coefficient of AtFlot1 (Figures 7A,C,E). Further, comparison AtFlot1 that the changes in diffusion coefficients and the velocity of AtHIR1 were more pronounced in plants treated with ABA and NaCl (Figures 7B,D,F). Changes in the dwell time of AtFlot1 and AtHIR1 under ABA and NaCl treatment were consistent with the changes in diffusion characteristics (Figure 7H). Plausible explanations for the shift in lateral mobility include changes in interactions with other proteins and confinement to less-mobile membrane domains; alternatively, a combination of these two factors within the PM may influence activation of signal transduction (Bucherl et al., 2017). Upon considering our experimental results, we concluded that spatial separation of AtFlot1 and AtHIR1 signaling platforms is crucial for the executing of abiotic stress signaling.

Our single-molecule analysis has provided novel information regarding the dynamics of PM nanodomain organization with unprecedented spatial and temporal resolution. We found, using SPT and FCS analyses, that the distribution of AtFlot1 and AtHIR1 is regulated by biotic and abiotic stress-induced regulatory signals. SPT coupled with FRAP demonstrated that environment stimuli regulate plant domain dynamics; the spatiotemporal dynamics of nanodomains clearly play a role in signaling in response to different stimuli. Together, our data suggest that the distinct spatiotemporal localization AtFlot1- and AtHIR1-containing nanodomains may contribute to signaling specificity by these nanodomains.

## Materials and Methods

### Plant Materials and Construction

Seeds were surface-sterilized with diluted commercial bleach for 10 min and sown on plates with half strength MS (Duchefa, Haarlem, Netherlands) medium solidified with 1% agar and supplemented with 1% sucrose. Seedlings were grown in the vertical position in 16/8 h light (100 μmol m^–2^⋅s^–1^)/dark cycles. Plants at 6-days old were used for the analysis of roots, cotyledons and hypocotyls, and plants at 10-days old were used in analyses of first true leaves.

### Drug Treatments

Drugs were used at the following concentrations: 10 μM Pep1 (10 mM stock in distilled deionized H_2_O), 10 μM flg22 (10 mM stock in distilled deionized H_2_O), 10 μM ABA (10 mM stock in distilled deionized H_2_O), and 100 mM NaCl. GFP-AtFlot1 and AtHIR1-GFP seedlings were treated with various working solutions for the durations described in each figure legend.

### Variable-Angle Total Internal Reflection Fluorescence Microscopy and Single-Particle Tracking Analyses

Seedlings were observed under VA-TIRFM with a × 100 oil-immersion objective (Olympus; numerical aperture = 1.45) (Cui et al., 2021). GFP-AtFlot1 and AtHIR1-GFP signals upon excitation at 488 nm wavelength using a diode laser (Changchun New Industries Optoelectronics Technology) were obtained with a BA510IF filter (525/50). A back-illuminated electron-multiplying charge-coupled device (EMCCD) camera (ANDOR iXon DV8897D-CS0-VP, Andor Technology) was used to detect fluorescent proteins, and the signals were stored on a computer. SPT analysis was performed according to the method described by Cui et al. (2018b). The dynamic and single-particle fluorescence intensities were measured as previously described (Wang et al., 2015).

### Fluorescence Recovery After Photobleaching Analysis

An FV1000MPE multiphoton laser-scanning microscope (Olympus) was used to perform FRAP experiments. The square region of interest was drawn and bleached with a 488 nm laser at 100% laser power. The time interval for monitoring fluorescence recovery was 3 s. The fluorescence recovery was quantified using Image J software (National Institutes of Health). The data obtained were corrected for bleaching during imaging as described by Xing et al. (2019). Origin 8.6 software (Origin Lab Corporation) was used for curve fitting.

### Fluorescence Correlation Spectroscopy Analysis

Fluorescence correlation spectroscopy analysis was conducted in point scanning mode using a Leica TCS SP5 FCS microscope equipped with a 488 nm argon laser, a coupled correlator built in-house, and an avalanche photodiode. The laser was focused on selected areas of the PM. The diffusion of GFP-AtFlot1 and AtHIR1-GFP molecules into and out of the focal volume altered the local concentration of fluorophores and led to spontaneous fluctuations in fluorescence intensity. Therefore, the GFP-AtFlot1 and AtHIR1-GFP density estimated in the confocal volume could be expressed as the total GFP-AtFlot1 or AtHIR1-GFP fluorescent signal divided by the area covered.

The GFP-AtFlot1 and AtHIR1-GFP density was determined in each individual cell membrane. Two random positions were selected once, and an autocorrelation measurement was performed for 20 s each for every measurement point. Up to six random points were selected in one cell, three cells were chosen in a leaf, and at least five representative leaves were studied for each measurement (Li et al., 2016).

### Data Analysis

The significance of arithmetic mean values for all data sets was assessed using Student’s *t*-test. Error bars were calculated with the s.d. function in Microsoft Excel. The differences at *P* < 0.05 were considered statistically significant. According to Student’s *t*-test, characters in the figure represent statistically significant differences compared with control (*P < 0.05, ^**^P < 0.01 and ^***^P < 0.001).

## Data Availability Statement

The original contributions presented in the study are included in the article/Supplementary Material, further inquiries can be directed to the corresponding authors.

## Author Contributions

JL, YC, and MY designed the research. YC, CX, and SA performed the research. YC, XZ, and XL contributed new reagents and analytic tools. YC, CX, and HQ analyzed the data. YC and JL wrote the manuscript. All authors contributed to the article and approved the submitted version.

## Conflict of Interest

The authors declare that the research was conducted in the absence of any commercial or financial relationships that could be construed as a potential conflict of interest.

## Publisher’s Note

All claims expressed in this article are solely those of the authors and do not necessarily represent those of their affiliated organizations, or those of the publisher, the editors and the reviewers. Any product that may be evaluated in this article, or claim that may be made by its manufacturer, is not guaranteed or endorsed by the publisher.

## References

[B1] Bucherl C. A. Jarsch I. K. Schudoma C. Segonzac M. bengue M. Robatzek S. (2017). Plant immune and growth receptors share common signalling components but localise to distinct plasma membrane nanodomains. *eLife* 6:e25114. 10.7554/eLife.25114 28262094 PMC5383397

[B2] Checker V. G. Khurana P. (2013). Molecular and functional characterization of mulberry EST encoding remorin (MiREM) involved in abiotic stress. *Plant Cell Rep.* 32 1729–1741. 10.1007/s00299-013-1483-5 23942844

[B3] Cui Y. N. Li X. J. Yu M. Li R. L. Fan L. S. Zhu Y. F. (2018a). Sterols regulate endocytic pathways during flg22-induced defense responses in *Arabidopsis*. *Development* 145:dev165688. 10.1242/dev.165688 30228101

[B4] Cui Y. N. Yu M. Yao X. M. Xing J. J. Lin J. X. Li X. J. (2018b). Single-particle tracking for the quantification of membrane protein dynamics in living plant cells. *Mol. Plant* 11 1315–1327. 10.1016/j.molp.2018.09.008 30296600

[B5] Cui Y. N. Zhang X. Yu M. Zhu Y. F. Xing J. J. Lin J. X. (2019). Techniques for detecting protein-protein interactions in living cells: principles, limitations, and recent progress. *Sci. China Life Sci.* 62 619–632. 10.1007/s11427-018-9500-7 30877434

[B6] Cui Y. N. Zhao Y. X. Lu Y. Q. Su X. Chen Y. Y. Shen Y. B. (2021). In vivo single-particle tracking of the aquaporin AtPIP2;1 in stomata reveals cell type-specific dynamics. *Plant Physiol.* 185 1666–1681. 10.1093/plphys/kiab007 33569600 PMC8133650

[B7] Danek M. Angelini J. Malinska K. Andrejch J. Amlerova Z. Kocourkova D. (2020). Cell wall contributes to the stability of plasma membrane nanodomain organization of Arabidopsis thaliana Flotillin2 and Hypersensitive Induced reaction1 proteins. *Plant J.* 101 619–636. 10.1111/tpj.14566 31610051

[B8] Danek M. Valentova O. Martinec J. (2016). Flotillins, erlins, and HIRs: from animal base camp to plant new horizons. *Crit. Rev. Plant Sci.* 35 191–214. 10.1080/07352689.2016.1249690

[B9] Danquah A. de Zelicourt A. Colcombet J. Hirt H. (2014). The role of ABA and MAPK signaling pathways in plant abiotic stress responses. *Biotechnol. Adv.* 32 40–52. 10.1016/j.biotechadv.2013.09.006 24091291

[B10] Diaz-Rohrer B. B. Levental K. R. Simons K. Levental I. (2014). Membrane raft association is a determinant of plasma membrane localization. *Proc. Natl. Acad. Sci. U.S.A.* 111 8500–8505. 10.1073/pnas.1404582111 24912166 PMC4060687

[B11] El Yahyaoui F. Kuster H. Ben Amor B. Hohnjec N. Puhler A. Becker A. (2004). Expression profiling in medicago truncatula identifies more than 750 genes differentially expressed during nodulation, including many potential regulators of the symbiotic program. *Plant Physiol.* 136 3159–3176. 10.1104/pp.104.043612 15466239 PMC523376

[B12] Flores-Otero J. Ahn K. H. Delgado-Peraza F. Mackie K. Kendall D. A. Yudowski G. A. (2014). Ligand-specific endocytic dwell times control functional selectivity of the cannabinoid receptor 1. *Nat. Commun.* 5:4589. 10.1038/ncomms5589 25081814 PMC4227836

[B13] Gong Z. Z. Xiong L. M. Shi H. Z. Luis R. H. Xu G. H. (2020). Plant abiotic stress response and nutrient use efficiency. *Sci. China Life Sci.* 63 635–674. 10.1007/s11427-020-1683-x 32246404

[B14] Grosjean K. Der C. Robert F. Thomas D. Mongrand S. Simon-Plas F. (2018). Interactions between lipids and proteins are critical for organization of plasma membrane-ordered domains in tobacco BY-2 cells. *J. Exp. Bot.* 69 3545–3557. 10.1093/jxb/ery152 29722895 PMC6022670

[B15] Haney C. H. Long S. R. (2010). Plant flotillins are required for infection by nitrogen-fixing bacteria. *Proc. Natl. Acad. Sci. U.S.A.* 107 478–483. 10.1073/pnas.0910081107 20018678 PMC2806772

[B16] Hao H. Q. Fan L. S. Chen T. Li R. L. Li X. J. He Q. H. (2014). Clathrin and membrane microdomains cooperatively regulate RbohD dynamics and activity in *Arabidopsis*. *Plant Cell* 26 1729–1745. 10.1105/tpc.113.122358 24755455 PMC4036582

[B17] Hemsley P. A. Weimar T. Lilley K. S. Dupree P. Grierson C. S. (2013). A proteomic approach identifies many novel palmitoylated proteins in *Arabidopsis*. *New Phytol.* 197 805–814. 10.1111/nph.12077 23252521

[B18] Hirayama T. Shinozaki K. (2010). Research on plant abiotic stress responses in the post-genome era: past, present and future. *Plant J.* 61 1041–1052. 10.1111/j.1365-313x.2010.04124.x 20409277

[B19] Jarsch I. K. Konrad S. S. A. Stratil T. F. Urbanus S. L. Szymanski W. Braun P. (2014). Plasma membranes are subcompartmentalized into a plethora of coexisting and diverse microdomains in *Arabidopsis* and *Nicotiana benthamiana*. *Plant Cell* 26 1698–1711. 10.1105/tpc.114.124446 24714763 PMC4036580

[B20] Laude A. J. Prior I. A. (2004). Plasma membrane microdomains: organization, function and trafficking (review). *Mol. Membr. Biol.* 21 193–205. 10.1080/09687680410001700517 15204627 PMC3376445

[B21] Li K. Du Y. L. Miao Y. C. (2016). Future challenges in understanding ROS in plant responses to abiotic stress. *Sci. China Life Sci.* 59 1343–1344. 10.1007/s11427-016-0362-7 27933592

[B22] Li L. Yu Y. F. Zhou Z. Y. Zhou J. M. (2016). Plant pattern-recognition receptors controlling innate immunity. *Sci. China Life Sci.* 59 878–888. 10.1007/s11427-016-0115-2 27535423

[B23] Li R. L. Liu P. Wan Y. L. Chen T. Wang Q. L. Mettbach U. (2012). A membrane microdomain-associated protein, *Arabidopsis* Flot1, is involved in a clathrin-independent endocytic pathway and is required for seedling development. *Plant Cell* 24 2105–2122. 10.1105/tpc.112.095695 22589463 PMC3442590

[B24] Li S. S. Zhao J. P. Zhai Y. S. Yuan Q. Zhang H. H. Wu X. Y. (2019). The hypersensitive induced reaction 3 (HIR3) gene contributes to plant basal resistance via an EDS1 and salicylic acid-dependent pathway. *Plant J.* 98 783–797. 10.1111/tpj.14271 30730076

[B25] Li X. J. Xing J. J. Qiu Z. B. He Q. H. Lin J. X. (2016). Quantification of membrane protein dynamics and interactions in plant cells by fluorescence correlation spectroscopy. *Mol. Plant* 9 1229–1239. 10.1016/j.molp.2016.06.017 27381442

[B26] Lv X. Q. Jing Y. P. Xiao J. W. Zhang Y. D. Zhu Y. F. Julian R. (2017). Membrane microdomains and the cytoskeleton constrain AtHIR1 dynamics and facilitate the formation of an AtHIR1-associated immune complex. *Plant J.* 90 3–16. 10.1111/tpj.13480 28081290

[B27] Majeran W. Caer J. Ponnala L. Meinnel T. Giglione C. (2018). Targeted profiling of *Arabidopsis* thaliana sub-proteomes illuminates new co- and post-translationally n-terminal myristoylated proteins. *Plant Cell* 30 543–562. 10.1105/tpc.17.00523 29453228 PMC5894833

[B28] Malinsky J. Opekarova M. Grossmann G. Tanner W. (2013). Membrane microdomains, rafts, and detergent-resistant membranes in plants and fungi. *Annu. Rev. Plant Biol.* 64 501–529. 10.1146/annurev-arplant-050312-120103 23638827

[B29] Mattson M. P. (2005). *Membrane Microdomain Signaling: Lipid Rafts In Biology And Medicine.* Totowa: Humana Press.

[B30] Morel J. Claverol S. Mongrand S. Furt F. Fromentin J. Bessoule J. J. (2006). Proteomics of plant detergent-resistant membranes. *Mol. Cell Proteomics* 5 1396–1411. 10.1074/mcp.M600044-MCP200 16648627

[B31] Owen D. M. Williamson D. Rentero C. Gaus K. (2009). Quantitative microscopy: protein dynamics and membrane organisation. *Traffic* 10 962–971. 10.1111/j.1600-0854.2009.00908.x 19416480

[B32] Qi Y. P. Tsuda K. Nguyen L. V. Wang X. Lin J. S. Murphy A. S. (2011). Physical association of Arabidopsis hypersensitive induced reaction proteins (HIRs) with the immune receptor RPS2. *J. Biol. Chem.* 286 31297–31307. 10.1074/jbc.M110.211615 21757708 PMC3173095

[B33] Shen W. W. Ma L. Y. Zhang X. Li X. X. Zhao Y. Y. Jing Y. P. (2020). Three-dimensional reconstruction of Picea wilsonii Mast. pollen grains using automated electron microscopy. *Sci. China Life Sci.* 63 171–179. 10.1007/s11427-019-9820-4 31625022

[B34] Smal I. Grigoriev I. Akhmanova A. Niessen W. J. Meijering E. (2010). Microtubule dynamics analysis using kymographs and variable-rate particle filters. *Ieee T Image Process.* 19 1861–1876. 10.1109/tip.2010.2045031 20227980

[B35] Su B. D. Zhang X. Li L. Abbas S. Yu M. Cui Y. N. (2021). Dynamic spatial reorganization of BSK1 complexes in the plasma membrane underpins signal-specific activation for growth and immunity. *Mol. Plant* 14 588–603. 10.1016/j.molp.2021.01.019 33524551

[B36] Tanner W. Malinsky J. Opekarova M. (2011). In plant and animal cells, detergent-resistant membranes do not define functional membrane rafts. *Plant Cell* 23 1191–1193. 10.1105/tpc.111.086249 21531862 PMC3101544

[B37] Vieira F. S. Correa G. Einicker-Lamas M. Coutinho-Silva R. (2010). Host-cell lipid rafts: a safe door for micro-organisms? *Biol. Cell* 102 391–407. 10.1042/bc20090138 20377525 PMC7161784

[B38] Wang L. Li H. Lv X. Q. Chen T. Li R. L. Xue Y. Q. (2015). Spatiotemporal dynamics of the BRI1 receptor and its regulation by membrane microdomains in living Arabidopsis cells. *Mol. Plant* 8 1334–1349. 10.1016/j.molp.2015.04.005 25896454

[B39] Xing J. J. Li X. J. Wang X. H. Lv X. Q. Wang L. Zhang L. (2019). Secretion of phospholipase Dδ delta functions as a regulatory mechanism in plant innate immunity. *Plant Cell* 31 3015–3032. 10.1105/tpc.19.00534 31597687 PMC6925013

[B40] Yu M. Cui Y. N. Zhang X. Li R. L. Lin J. X. (2020a). Organization and dynamics of functional plant membrane microdomains. *Cell Mol. Life Sci.* 77 275–287. 10.1007/s00018-019-03270-7 31422442 PMC11104912

[B41] Yu M. Li R. L. Cui Y. N. Chen W. Li B. Zhang X. (2020b). The RALF1-FERONIA interaction modulates endocytosis to mediate control of root growth in *Arabidopsis*. *Development* 147:dev189902. 10.1242/dev.189902 32541006

[B42] Yu M. Liu H. J. Dong Z. Y. Xiao J. W. Su B. D. Fan L. S. (2017). The dynamics and endocytosis of Flot1 protein in response to flg22 in *Arabidopsis*. *J. Plant Physiol.* 215 73–84. 10.1016/j.jplph.2017.05.010 28582732

[B43] Zhang Q. Li Y. L. Tsien R. W. (2009). The dynamic control of kiss-and-run and vesicular reuse probed with single nanoparticles. *Science* 323 1448–1453. 10.1126/science.1167373 19213879 PMC2696197

[B44] Zhang X. Cui Y. N. Yu M. Su B. D. Gong W. Baluska F. (2019). Phosphorylation-mediated dynamics of nitrate transceptor NRT1.1 regulate auxin flux and nitrate signaling in lateral root growth. *Plant Physiol.* 181 480–498. 10.1104/pp.19.00346 31431511 PMC6776865

[B45] Zhou J. M. Yang W. C. (2016). Receptor-like kinases take center stage in plant biology. *Sci. China Life Sci.* 59 863–866. 10.1007/s11427-016-5112-8 27604522

[B46] Zhou J. M. Zhang Y. L. (2020). Plant immunity: danger perception and signaling. *Cell* 181 978–989. 10.1016/j.cell.2020.04.028 32442407

[B47] Zhu D. M. Zhang M. D. Gao C. J. Shen J. B. (2020). Protein trafficking in plant cells: Tools and markers. *Sci. China Life Sci.* 63 343–363. 10.1007/s11427-019-9598-3 31707539

[B48] Zipfel C. Oldroyd G. E. D. (2017). Plant signalling in symbiosis and immunity. *Nature* 543 328–336. 10.1038/nature22009 28300100

