# Supplementary material for: Environmental Cues Contribute to Dynamic Plasma Membrane Organization of Nanodomains Containing Flotillin-1 and Hypersensitive Induced Reaction-1 Proteins in Arabidopsis thaliana
Source: Front Plant Sci. 2022 May 10;13:897594. doi: 10.3389/fpls.2022.897594 (PMC9127874; doi:10.3389/fpls.2022.897594)
Supplement: Supplementary file 1 [file Data_Sheet_1.docx]

Environmental cues contribute to dynamic plasma membrane nanodomain organization of *Arabidopsis thaliana* Flotillin1 and hypersensitive induced Reaction1 proteins

Changwen Xu, Sammar Abbas, Hongping Qian, Meng Yu, Xi Zhang, Xiaojuan Li, Yaning Cui, Jinxing Lin


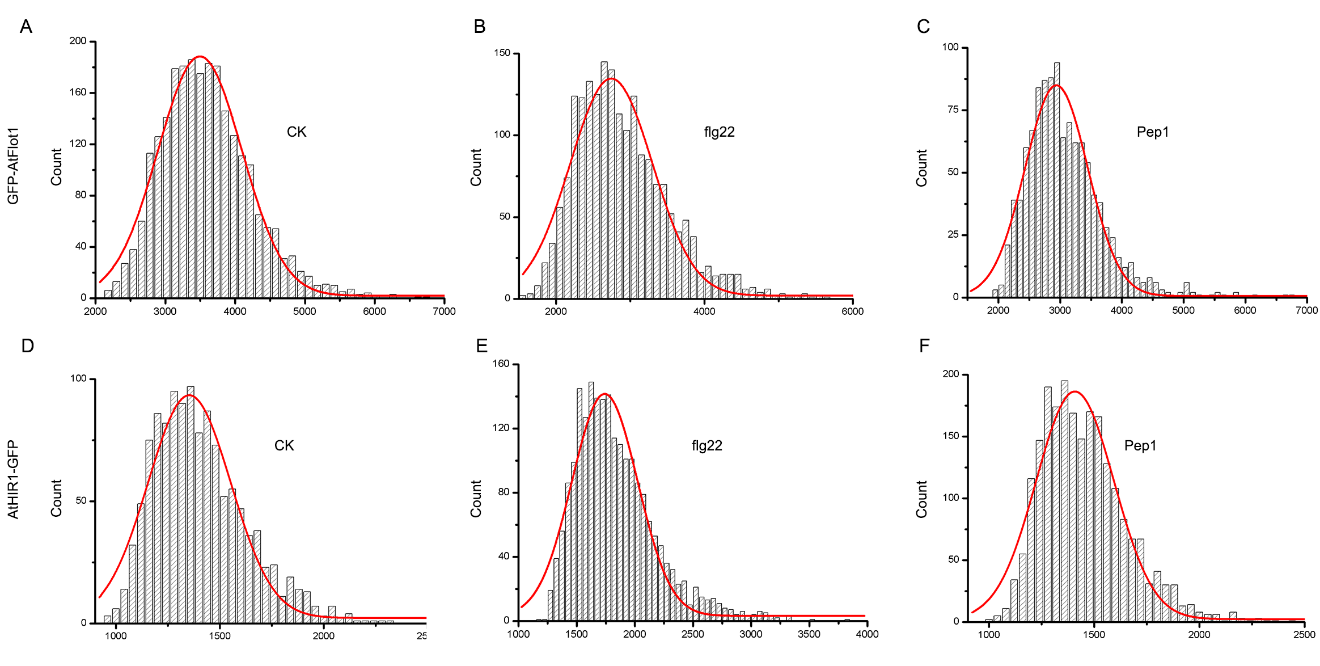


Fig. S1 Effects of flg22 and Pep1 on the intensity of GFP-AtFlot1 and AtHIR1-GFP spots in Arabidopsis thaliana.

(A) Distribution of GFP-AtFlot1 intensity under control.

(B) Distribution of GFP-AtFlot1 intensity under flg22 treatment.

(C) Distribution of GFP-AtFlot1 intensity under Pep1 treatment.

(D) Distribution of AtHIR1-GFP intensity under control.

(E) Distribution of AtHIR1-GFP intensity under flg22 treatment.

(F) Distribution of AtHIR1-GFP intensity under Pep1 treatment.


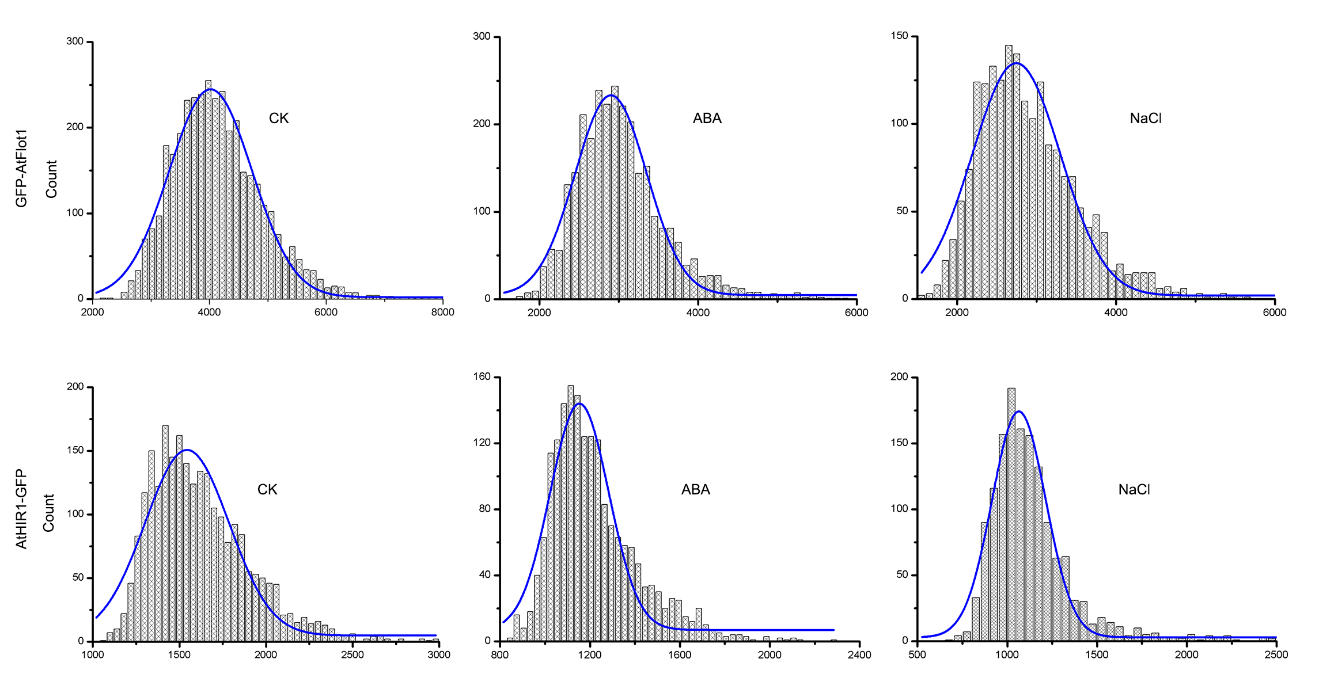


Fig. S2 Effects of ABA and NaCl on the intensity of GFP-AtFlot1 and AtHIR1-GFP spots in Arabidopsis thaliana.

(A) Distribution of GFP-AtFlot1 intensity under control.

(B) Distribution of GFP-AtFlot1 intensity under ABA treatment.

(C) Distribution of GFP-AtFlot1 intensity under NaCl treatment.

(D) Distribution of AtHIR1-GFP intensity under control.

(E) Distribution of AtHIR1-GFP intensity under ABA treatment.

(F) Distribution of AtHIR1-GFP intensity under NaCl treatment.


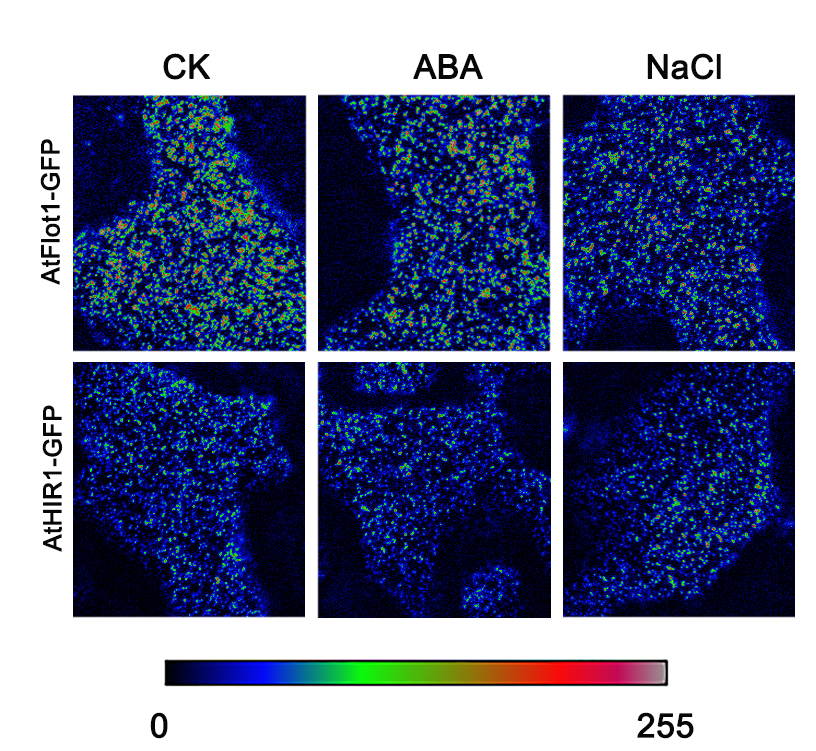


Fig. S3 Pseudocolor images (blue-yellow-red palette) show the fluorescence intensity of GFP-AtFlot1 and AtHIR1-GFP at the plasma membrane (color code bar indicates the

relative intensities). Bar = 10 μm

Fig. S
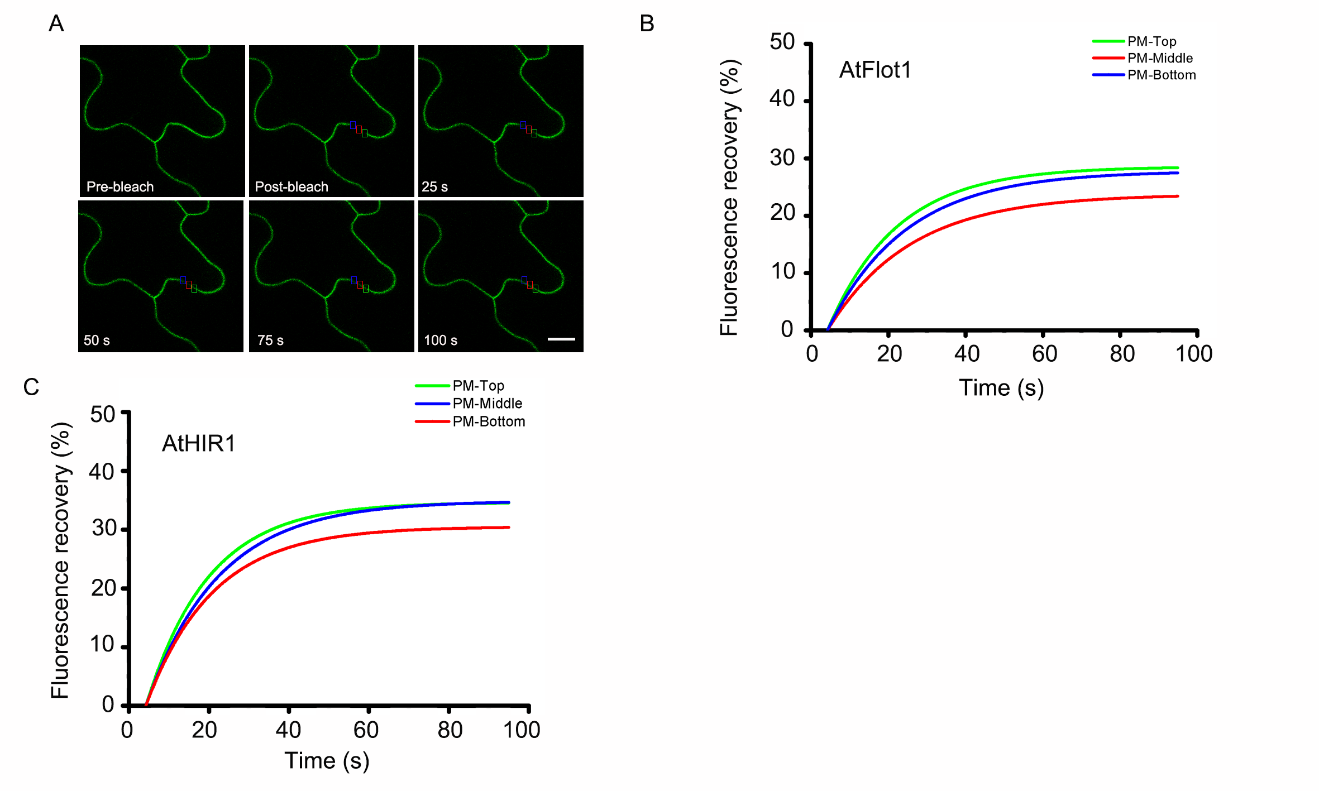
4 FRAP analysis revealing the dynamics of GFP-AtFlot1 and AtHIR1-GFP.

(A) Timecourse of FRAP of GFP. The initial bleaching region was subdivided into three sectors, as indicated by the red, green and blue squares. Time is given in seconds.

(B) Fluorescence recovery curves of divided regions of interest. Curves represent the best fits of mean values of six independent FRAP experiments on GFP-AtFlot1.

(C) Fluorescence recovery curves of divided regions of interest. Curves represent the best fits of mean values of six independent FRAP experiments on AtHIR1-GFP.

Fig. S
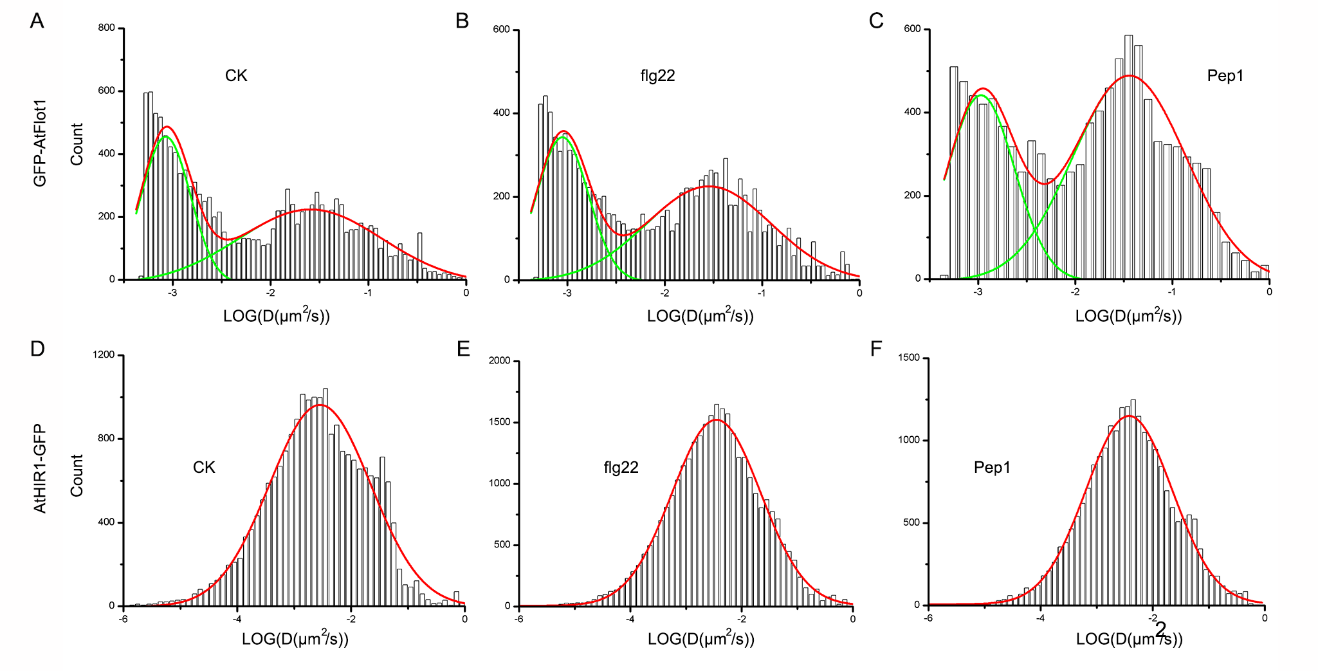
5 Effects of flg22 and Pep1 on the diffusion coefficients of GFP-AtFlot1 and AtHIR1-GFP spots.

(A) Distribution of GFP-AtFlot1 diffusion coefficients under control.

(B) Distribution of GFP-AtFlot1 diffusion coefficients under flg22 treatment.

(C) Distribution of GFP-AtFlot1 diffusion coefficients under Pep1 treatment.

(D) Distribution of AtHIR1-GFP diffusion coefficients under control.

(E) Distribution of AtHIR1-GFP diffusion coefficients under flg22 treatment.

(F) Distribution of AtHIR1-GFP diffusion coefficients under Pep1 treatment.

Fig. S
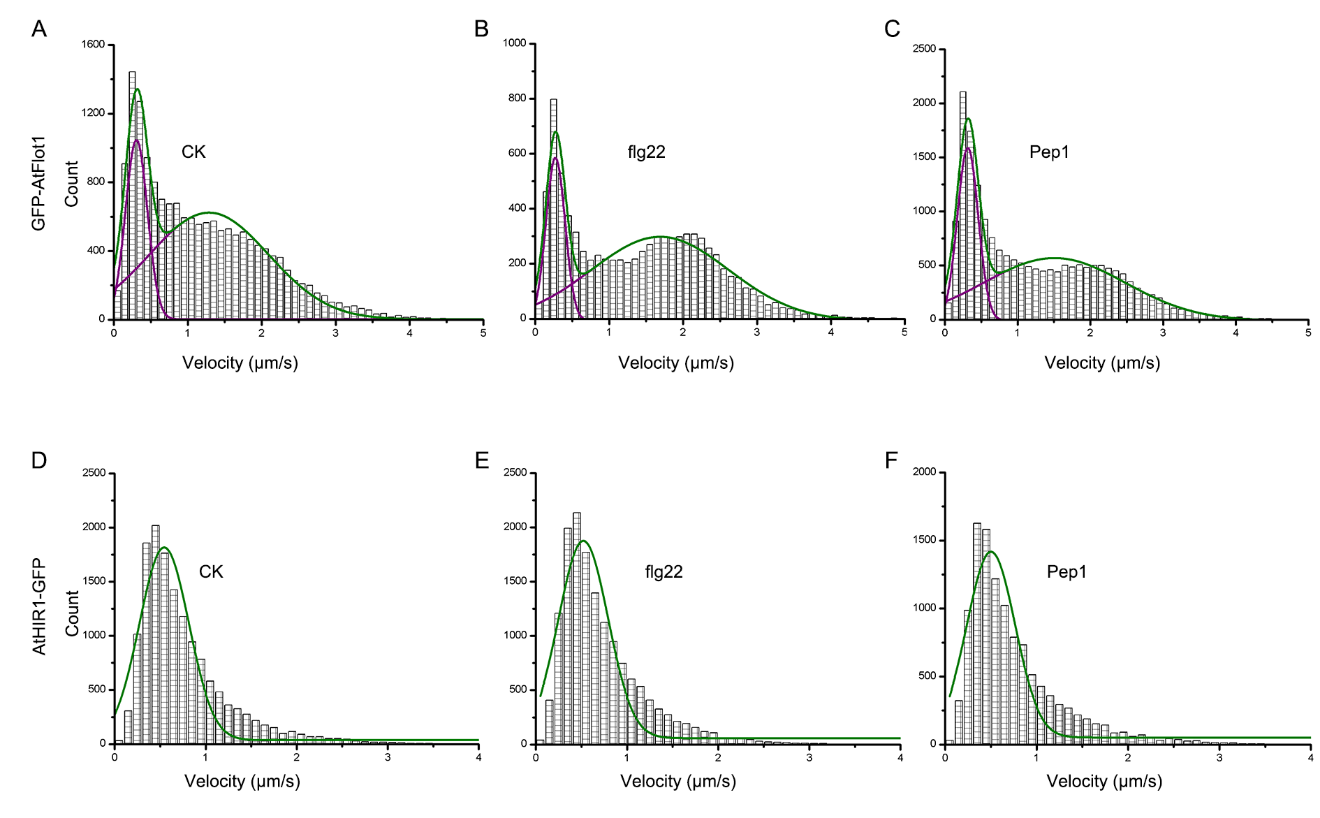
6 Effects of flg22 and Pep1 on the velocity of GFP-AtFlot1 and AtHIR1-GFP spots.

(A) Distribution of GFP-AtFlot1 velocity under control.

(B) Distribution of GFP-AtFlot1 velocity under flg22 treatment.

(C) Distribution of GFP-AtFlot1 velocity under Pep1 treatment.

(D) Distribution of AtHIR1-GFP velocity under control.

(E) Distribution of AtHIR1-GFP velocity under flg22 treatment.

(F) Distribution of AtHIR1-GFP velocity under Pep1 treatment .


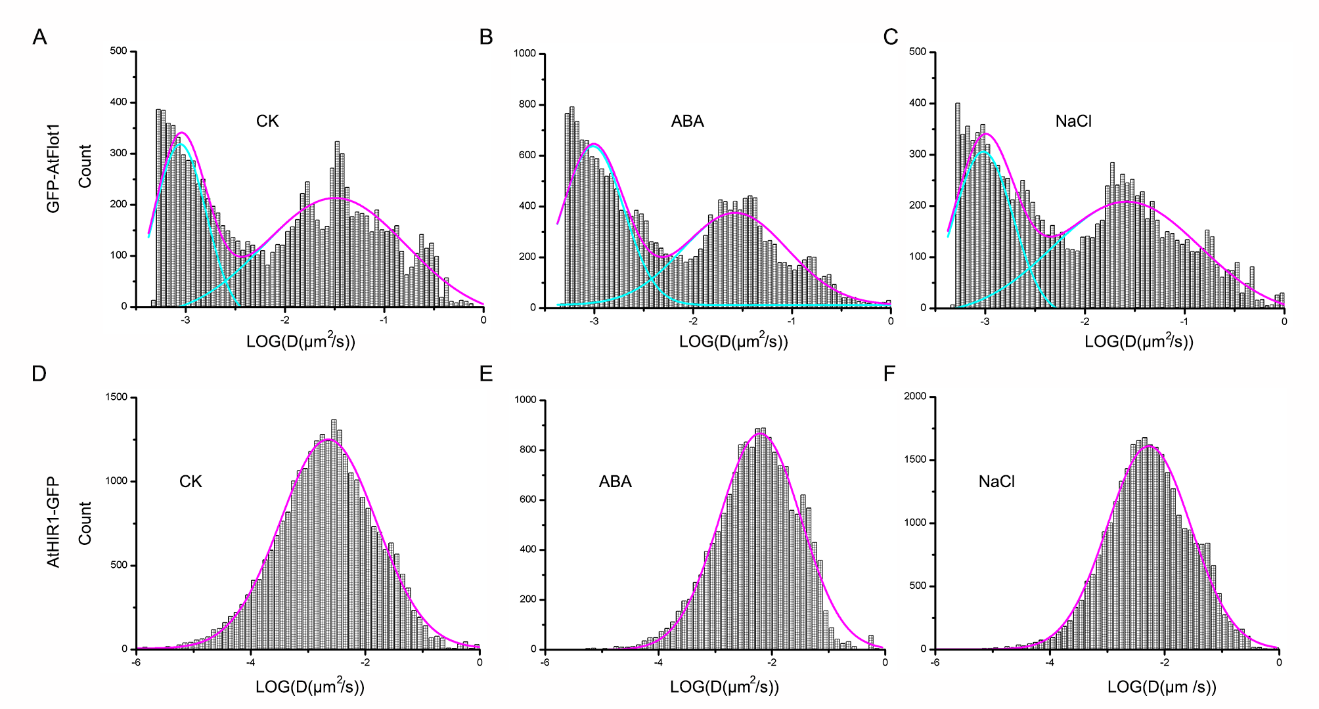
Fig. S7 Effects of ABA and NaCl on the diffusion coefficients of GFP-AtFlot1 and AtHIR1-GFP spots.

(A) Distribution of GFP-AtFlot1 diffusion coefficients under control.

(B) Distribution of GFP-AtFlot1 diffusion coefficients under ABA treatment.

(C) Distribution of GFP-AtFlot1 diffusion coefficients under NaCl treatment.

(D) Distribution of AtHIR1-GFP diffusion coefficients under control.

(E) Distribution of AtHIR1-GFP diffusion coefficients under ABA treatment.

(F) Distribution of AtHIR1-GFP diffusion coefficients under NaCl treatment.

Fig. S
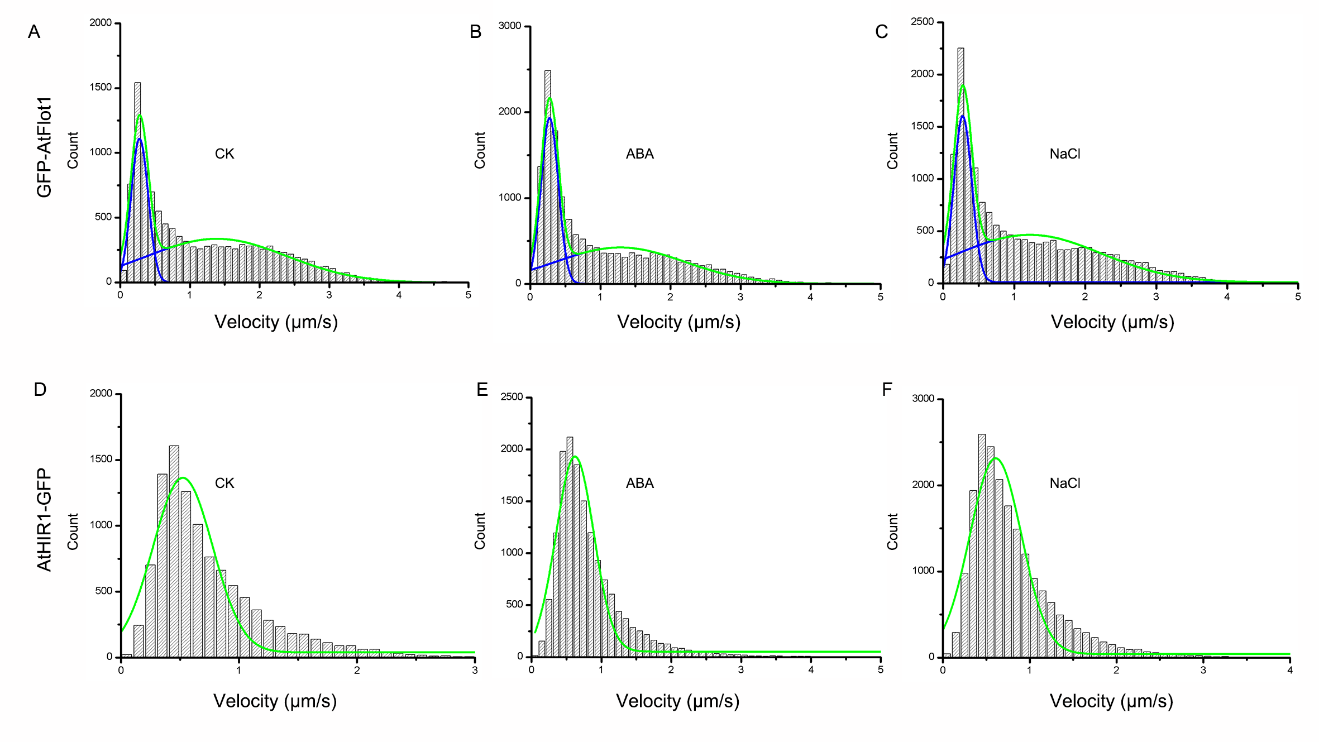
8 Effects of ABA and NaCl on the velocity of GFP-AtFlot1 and AtHIR1-GFP spots.

(A) Distribution of GFP-AtFlot1 velocity under control.

(B) Distribution of GFP-AtFlot1 velocity under ABA treatment.

(C) Distribution of GFP-AtFlot1 velocity under NaCl treatment.

(D) Distribution of AtHIR1-GFP velocity under control.

(E) Distribution of AtHIR1-GFP velocity under ABA treatment.

(F) Distribution of AtHIR1-GFP velocity under NaCl treatment.
